# Artesunate attenuates traumatic brain injury-induced impairments in rats

**DOI:** 10.1515/tnsci-2020-0136

**Published:** 2020-09-09

**Authors:** Zhike Zhou, Jun Hou, Qinghua Li

**Affiliations:** Department of Dermatology, Qingdao Municipal Hospital, No. 21 Anhui Road, Qingdao 266001, Shandong, China

**Keywords:** artesunate, traumatic brain injuries, blood–brain barrier, inflammation

## Abstract

**Background:**

Blood–brain barrier (BBB) dysfunction and neuroinflammation induced by traumatic brain injuries (TBIs) cause a succession of secondary brain damage events and finally lead to a massive and progressive cerebral neuronal destruction. Artesunate, a semisynthetic artemisinin derivative, is a potential candidate for the management of cerebral damage induced by TBI due to its protective function to BBB and cerebral neurons.

**Methods:**

To demonstrate the effect of artesunate to TBI-induced BBB dysfunction and neural damage, TBI rat model was constructed by cortical impact injury. Behavioral experiments were used to estimate the impact of the combined treatment on rats. Western blotting was performed to demonstrate the protein levels in the brain tissues of rats. Quantitative real-time PCRs were utilized to investigate the alteration in the expression of various RNA levels. The chemokine levels were estimated by ELISA.

**Results:**

Artesunate treatment attenuated the impact caused by TBI on rat brain and improved the long-term neurological recover. Artesunate treatment protected the integrity of BBB and inhibited neuroinflammation. Artesunate treatment promoted the phosphorylation of Akt and inhibited the phosphorylation of glycogen synthase kinase (GSK)-3β in TBI rat model.

**Conclusion:**

Artesunate protected rats from TBI-induced impairments of BBB and improved longer-term neurological outcomes.

## 1 Introduction

Traumatic brain injury (TBI) has been ranked as one of the leading causes of disability and death induced by trauma, and approximately 13 million patients in USA and Europe are estimated to be impaired by the premature physical disability as well as cognitive impairment [1,2]. The alteration in brain membrane structure, neuronal metabolism, cerebral blood flow and other physiological abnormalities caused by the TBI induces a succession of secondary brain damage events and finally lead to a massive and progressive cerebral neuronal destruction such as brain edema and intracranial hypertension [3,4,5].

Blood–brain barrier (BBB) is composed of compact joint cerebrovascular endothelial cells and the permeability of BBB plays a crucial role in maintaining the homeostasis of brain and systemic circulation [6,7,8]. Posttraumatic BBB damage affects the prognosis of TBI patient and leads to devastating consequences including endothelial oxidative stress, neuron inflammation, deviant membrane permeability and cerebral infections [9,10]. However, the therapeutic strategies targeting posttraumatic BBB dysfunction were rather limited [11].

Artesunate is a semisynthetic artemisinin derivative [12]. The novel antimalaria therapeutic strategies based on multiple derivatives of artemisinin were recommended by the World Health Organization (WHO) due to their higher safety and better toleration [13]. In recent years, accumulating research have revealed the strong cytotoxic and anti-inflammatory effect of artesunate and its comprehensive application in the management of multiple diseases including cancer, viruses and fungi infection, sepsis, hemorrhage, resuscitation and arthritis [14,15]. The ability of artesunate to penetrate the BBB and maintain a relative high concentration in cerebral tissues makes it a potential candidate for cerebral disease management [12]. It has been reported that artesunate treatment improves the neuronal survival rate by regulating the expression of the neurotrophic factors in TBI mice model [16]. And artesunate has also been revealed to protect the integrity of BBB from subarachnoid hemorrhage (SAH) through sphingosine-1-phosphate receptor-1 (S1P1) and phosphatidylinositol 3 kinase (PI3K) pathways and thus to improve the long-term neurological outcomes [17]. However, there is no former research focusing on the protection ability of artesunate to the TBI-induced BBB dysfunction and neurological damage.

Neuroinflammation is an important factor for long-term chronic brain damage caused by TBIs. In the pathological process of TBI-induced neuroinflammation, PI3K and Akt not only regulate the expression of some key inflammatory factors at the injury site but also directly regulate the activity of glycogen synthase kinase (GSK)-3β by phosphorylating GSK-3β [18]. And GSK-3β itself plays a critical role in glutamate uptake, glutamate receptor trafficking and synaptic neurotransmission of nerve cells [19]. Therefore, PI3K/Akt/GSK-3β signaling pathway plays a decisive role in neuroinflammation and recovery of normal neurological function after TBI [20].

In this study, we reported that artesunate treatment protected rats from TBI-induced impairments of BBB and improved longer term neurological outcomes based on the existing evidence. We demonstrated that artesunate treatment induced the decrease in brain water content and protected the integrity of BBB after traumatism. Artesunate inhibited the cerebral inflammatory through Akt and GSK-3β pathway and thus improved the long-term neurological outcomes of model rats.

## 2 Methods

### 2.1 Animals

The adult male Sprague-Dawley rats purchased from SLAC (Shanghai, China) were utilized to perform the research. All the rats were cultured in the AAALAC-approved animal facility with a virus-/antigen-free ventilation system and an air-conditioning system with constant temperature and humidity. The food and water for the rats were obtained free.

Cortical impact injury (CCI) was performed to induce the TBI in rats as described formerly. Two percent isoflurane with oxygen was applied to induce anesthesia in rats. Then the skin and periosteum of the rats along the brain midline were sliced until the parietal bone was exposed. A surgical drill was utilized to open a small hole of 3.5 mm behind the coronal suture, and 3 mm to the cerebral impact device was attached to it to execute successive pressure. The impact device executed a pressure with a depth of 3.2 mm for 0.5 s at 4.0 m/s velocity to the brain of rats. The same procedure without pressure destruction was performed in the rats of sham groups.

Artesunate (Corp. Ltd, Chongqing, China) was dissolved in 5% sodium bicarbonate solution (Holley Wulingshan Pharmaceuticals, Chongqing, China). Various dosages of artesunate (25, 50, 100 and 200 mg/kg) were given intraperitoneally to TBI rats once a day and 5% sodium bicarbonate solution was used as the negative control. The time flow of this experiment was shown in Figure S1. The day when the CCI operation was performed on the rat was regarded as Day 0. The day before CCI was regarded as Day −1 and the day after the CCI operation was regarded as Day 1 and so on. Therefore, the administration time of astaxanthin and sodium bicarbonate solution was from Day −7 to 21. The brain infarct volume essay, brain water content assay, BBB measurements, quantitative real-time PCR (qRT-PCR), ELISA and Western blotting were performed at Day 3. The foot-fault tests were performed at Days 0, 3, 7, 14 and 21. The Morris water maze (MWM) assay was performed from Days 17 to 20.


**Ethical approval:** The research related to animals use has been complied with all the relevant national regulations and institutional policies for the care and use of animals. All experiments and procedures performed in animals in this research were examined and approved by the animal use committee in Qingdao Municipal Hospital.

### 2.2 Brain water content measurement

Wet–dry method was used to measure the brain water content of TBI rats as described previously [21]. A 5-mm coronal cerebral section in the center of the impacted cerebral hemisphere was weighed ant its total weight was recorded. Then the tissue block was dried in a dryer under 80°C for 48 h. The weight of the dried tissue blocked was then recorded. And the brain water content was calculated as (wet weight − dry weight)/wet weight × 100%.

### 2.3 MWM assay

The rats were divided into three groups based on the CCI and artesunate treatment with 10 rats per group. The experiment lasted for 5 days and all rats were scheduled to be trained four times a day for a fixed period of time. During the training, the rats were placed in the pool through four inlet points. The time that the rats took to enter the water to find the underwater concealed platform and stand on it was recorded as the incubation period. The rats were maintained to stay on the platform if they could find it by themselves. If the rats failed to find the platform in 60 s, they were gently pulled onto the platform for 10 s. Each rat was placed in the pool through four water inlet points with a 30 s interval between the training sessions.

On the fifth day, the rats were placed in the water at the same water inlet point in each quadrant and the swimming path of the rats in 120 s was recorded. The number of times that the rats crossed the target quadrant platform was recorded and used to evaluate the spatial localization ability of the rats.

### 2.4 Foot-fault assay

The impact induced by TBI to the motor coordination of rats was estimated by foot-fault assay as described formerly[22]. An elevated clinic ladder was used to train the rats for 1 week before the procedure, and the average scores of 2 days before the CCI were adapted as the standard scores of each rat. The same train and test were performed every day after the CCI for 3 weeks. And the scores of the tests were identified as the average scores of three tests.

### 2.5 Western blotting

Cells were collected and the protein of the cells was extracted by radioimmunoprecipitation (RIPA) buffer (ATCC, Manassas, VA, USA). All the samples were mixed with a loading buffer and boiled for 10 min to denature the proteins; 10% sodium dodecyl sulfate–polyacrylamide gel electrophoresis was used to separate the proteins, and the nitrocellulose (NC) membranes (Invitrogen Life Technologies, Carlsbad, CA, USA) were applied for protein transition. Then the membranes were incubated with anti-claudin-5, anti-occludin, anti-Zo1, anti-Akt, anti-p-Akt, anti-GSK-3β, anti-p-GSK-3β, anti-MMP9 and anti-β-actin (Centennial, CO, USA), respectively, under 4°C overnight. Secondary antibodies (Life Technology) were then incubated with the membranes after the washing of PBST under 37°C for 1 h.

### 2.6 qRT-PCR

RNA from trophoblasts of the model mice was extracted by TRIzol regent (Invitrogen Life Technologies) under the introduction of instructions. The reverse transcription was then done with 2 µg RNA of each kind of cells and Reverse Transcription Kits (Fermentas, St. Leon-Rot, Germany) to produce complementary DNA (cDNA); 2 µg of cDNA and 0.5 µM of each primer were used to establish the 20 µL real-time PCR system, and the mixes were detected by S1000 PCR Thermal cycler. β-Actin was used as the endogenous control.

The primers used in this assay were shown as follows:

IL-1β: 5′-CTGTGACTCATGGGATGATGATG-3′, 5′-CGGAGCCTGTAGTGCAGTTG-3′; IL-6: 5′-TCTATACCACTTCACAAGTCGGA-3′, 5′-GAATTGCCATTGCACAACTCTTT-3′; TNF-α: 5′-CCTGTAGCCCACGTCGTAG-3′, 5′-GGGAGTAGACAAGGTACAACCC-3′; MMP-9: 5′-GCGTCGTGATCCCCACTTAC-3′, 5′-CAGGCCGAATAGGAGCGTC-3′; GAPDH: 5′-TGGCCTTCCGTGTTCCTAC-3′, 5′-GAGTTGCTGTTGAAGTCGCA-3′.

### 2.7 ELISA

The blood of rats that suffered TBIs was collected in an ELISA kit (Abcam, Shanghai, China) and the contents of TNF-α, IL-1β and IL-6 in the blood were estimated.

### 2.8 Statistical analysis

All data were examined by one- or two-way ANOVA with an appropriate *post hoc* test and shown as mean ± SD. *P* < 0.05, hinting a statistical significance in the divergence. The analysis and calculation were performed by SPSS 16.0 software (SPSS, Inc., Chicago, IL, USA).

## 3 Results

### 3.1 Artesunate-attenuated TBI

**Figure 1:**
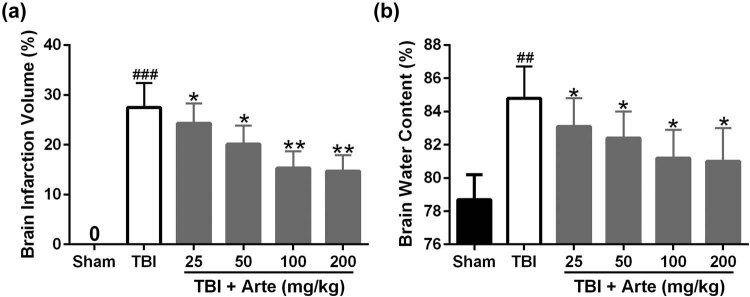
Artesunate-attenuated traumatic brain injury 3 days post-injury. Brain infarct volume (a) and brain water content (b) were measured after treatment with different dosages of artesunate (25, 50, 100, and 200 mg/kg). Data are presented as mean ± SD. *n* = 6 for each group. ^##^ *p* < 0.01 and ^###^ *p* < 0.001 compared to sham group. *p < 0.05 and **p < 0.01 compared to the TBI group.

To demonstrate the protective function of artesunate in TBI, magnetic resonance imaging (MRI) was used to estimate the brain infraction volume. As shown in Figure 1a, the brain infarction induced by TBI was attenuated significantly with increasing dosages of artesunate. Similarly, the TBI-induced brain water content also decreased dramatically with the increasing dosage of artesunate treatment (Figure 1b); 100 mg/kg artesunate shows a similar effect to the brain damage induced by TBI as 200 mg/kg, and we used 100 mg/kg artesunate for further experiments.

### 3.2 Artesunate improved long-term neurological outcomes after TBI

**Figure 2:**
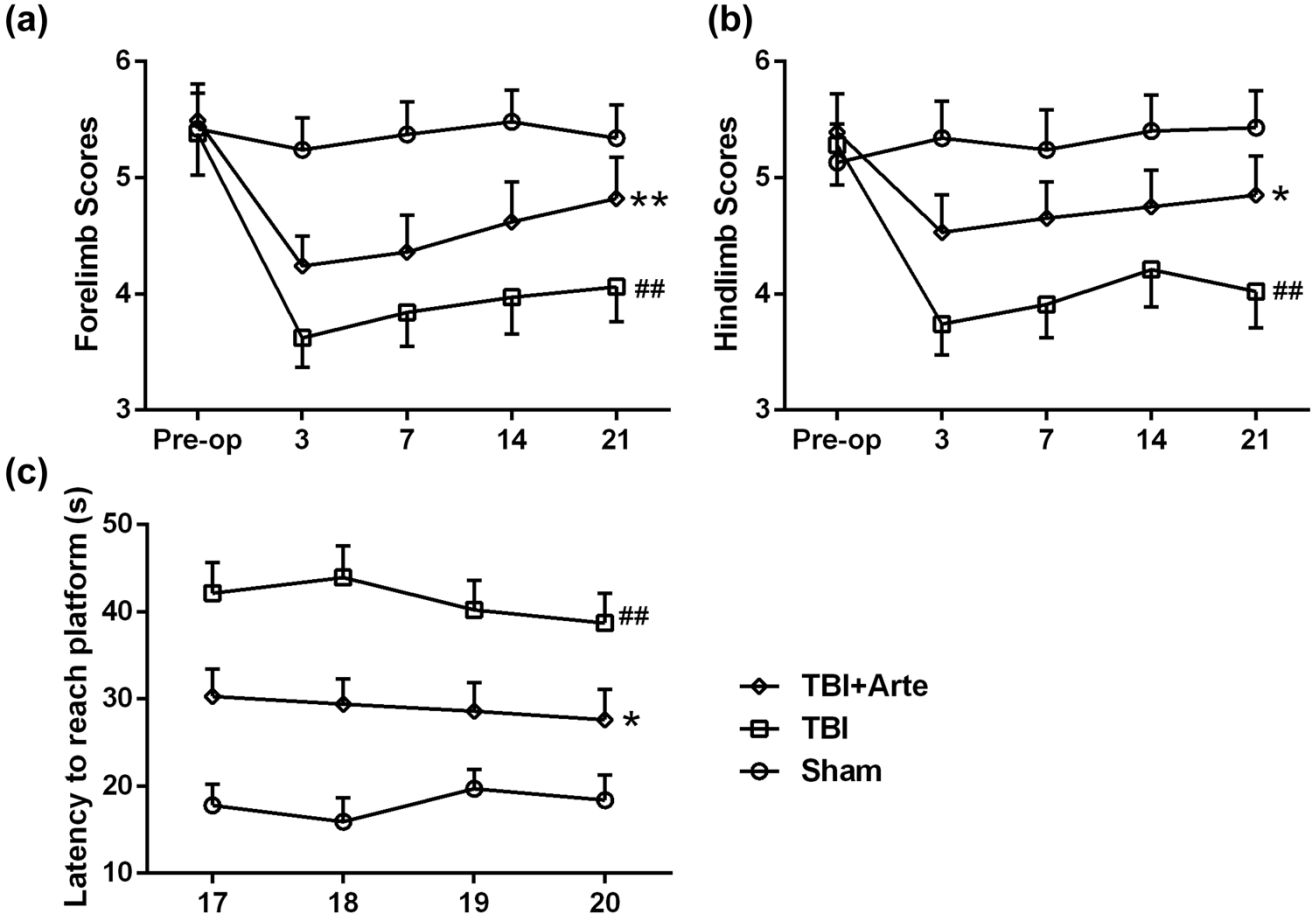
Artesunate improved the long-term neurological outcomes after traumatic brain injury. In the foot-fault tests, artesunate treatment (100 mg/kg) significantly improved the scores of left forelimbs (a) and hindlimb (b) compared to vehicle-treated TBI rats up to 3-week post-injury. “Pre-op” represents preinjury levels. In the Morris water maze tests, artesunate treatment (100 mg/kg) significantly shortened the escape latency from days 17 to 20 postinjury (c) compared to vehicle-treated TBI rats. Data are presented as mean ± SD. *n* = 8 for each group. ^##^ *p* < 0.01 compared to sham group. *p < 0.05, **p < 0.01 compared to TBI group.

To investigate the cerebral neural protective function of artesunate in the brain damage induced by TBI, behavior experiments of rat were performed. As shown in Figure 2a and b, artesunate treatment improved the limb-movement scores statistically in the foot-fault experiment. Besides, the MWM tests demonstrated that the artesunate treatment (100 mg/kg) significantly shortened the escape latency from days 17 to 20 post-injury compared to the vehicle-treated TBI rats (Figure 2c). These results suggested that artesunate improved long-term neurological outcomes after TBI.

### 3.3 Artesunate defended BBB integrity following TBI

**Figure 3:**
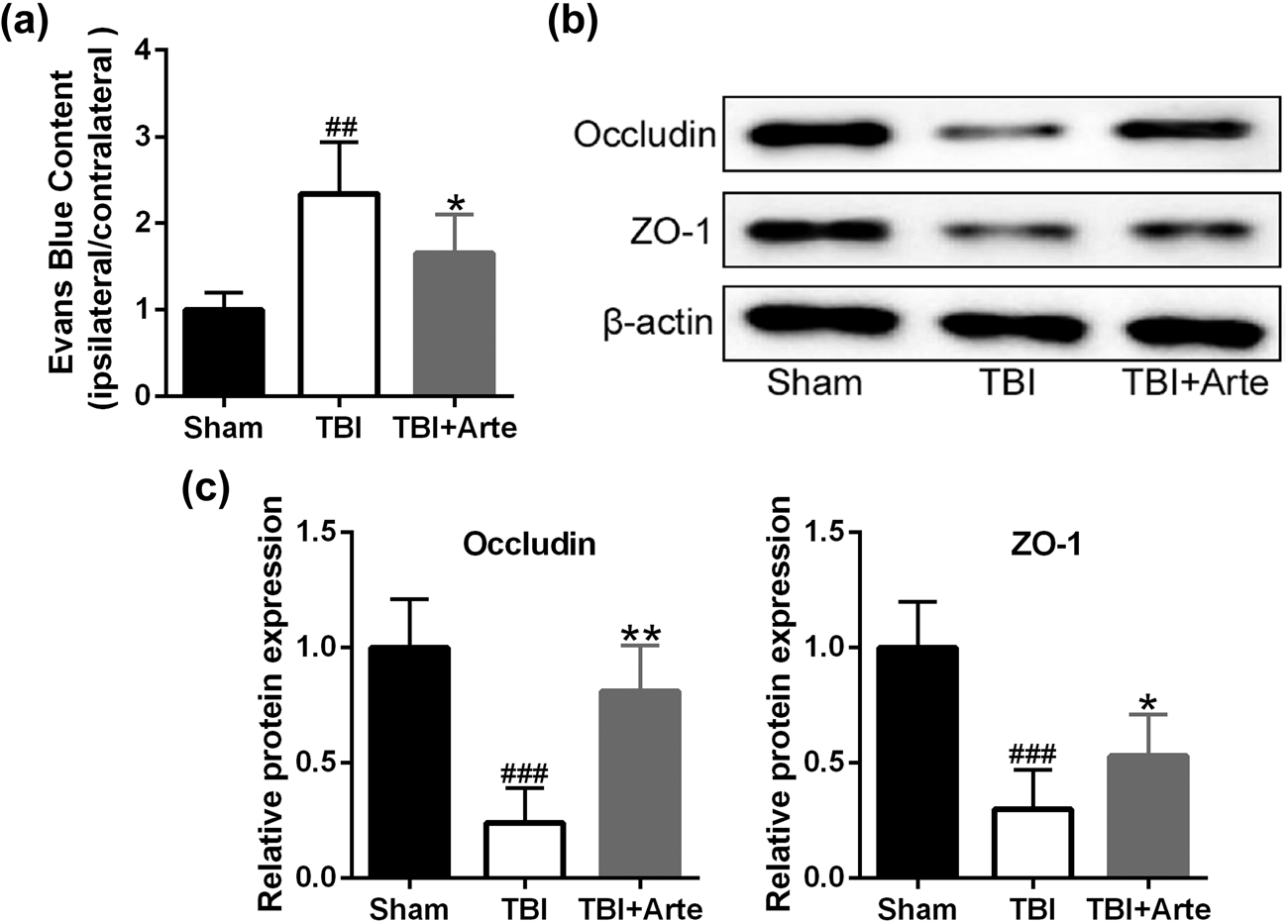
Artesunate defended BBB integrity following traumatic brain injury. (a) Evans blue extravasation was used to measure the blood–brain barrier (BBB) permeability 3 days post-injury. (b) Immunoblot analysis of junction proteins ZO-1 and occludin expressions in the ipsilateral brain tissue of experimental rats at 3 days post-injury. Quantitative analysis was normalized to sham (c). Data are presented as mean ± SD. *n* = 8 for each group. ^##^ *p* < 0.01 and ^###^ *p* < 0.001 compared to sham group. *p < 0.05 and **p < 0.01 compared to TBI group.

BBB integrity destruction was a common symptom in TBIs. To demonstrate that artesunate functioned to protect the BBB in TBI rats, Evans blue extravasation was used to measure the BBB permeability 3 days post-injury. As shown in Figure 3a, the blue content decreased with the artesunate treatment compared to the vehicle treatment, indicating lower permeability and higher integrity of BBB in artesunate-treated TBI rats. Occludin and Zo-1 played a significant role in the formation of the tight junction of epithelium in BBB [23]. As shown in Figure 3b and c, artesunate upregulated the protein levels of occludin and Zo-1 in TBI rats, indicating that artesunate protected the integrity of BBB.

#### 3.3.1 Artesunate inhibited TBI-induced MMP-9 expression in the ipsilateral brain

**Figure 4:**
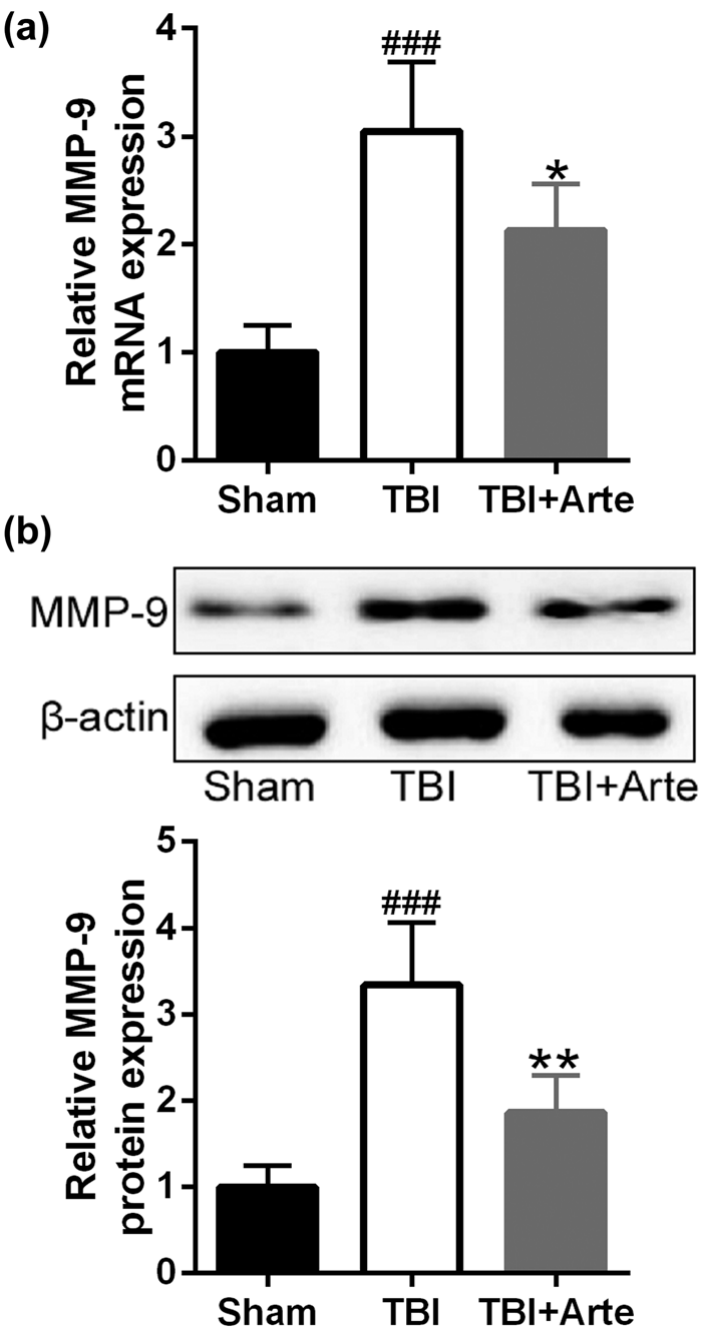
Artesunate inhibited traumatic brain injury-induced MMP-9 expression in the ipsilateral brain tissue of experimental rats 3 days post-injury. qRT-PCR was used to analyze the mRNA levels of MMP-9 (a) and Western blotting was used to measure the protein levels of MMP-9 (b). Data are presented as mean ± SD. *n* = 8 for each group. ^###^ *p* < 0.001 compared to sham group. *p < 0.05, **p < 0.01 compared to TBI group.

The upregulation of MMP-9 in the brain tissue was rather common in the TBI-induced neural damage, and the MMP-9 level has been used as an important marker to evaluate brain damage [24]. To further investigate the protective function of artesunate to TBI-induced brain damage, the RNA level of MMP-9 was first identified. As shown in Figure 4a, the RNA level of MMP-9 decreased dramatically in the artesunate-treated rats compared to the vehicle-treated rats. Similarly, artesunate also reduced the protein level of MMP-9 in TBI rats (Figure 4b), suggesting that artesunate did attenuate the brain damage in TBI rats.

### 3.4 Artesunate inhibited TBI-induced inflammatory response

**Figure 5:**
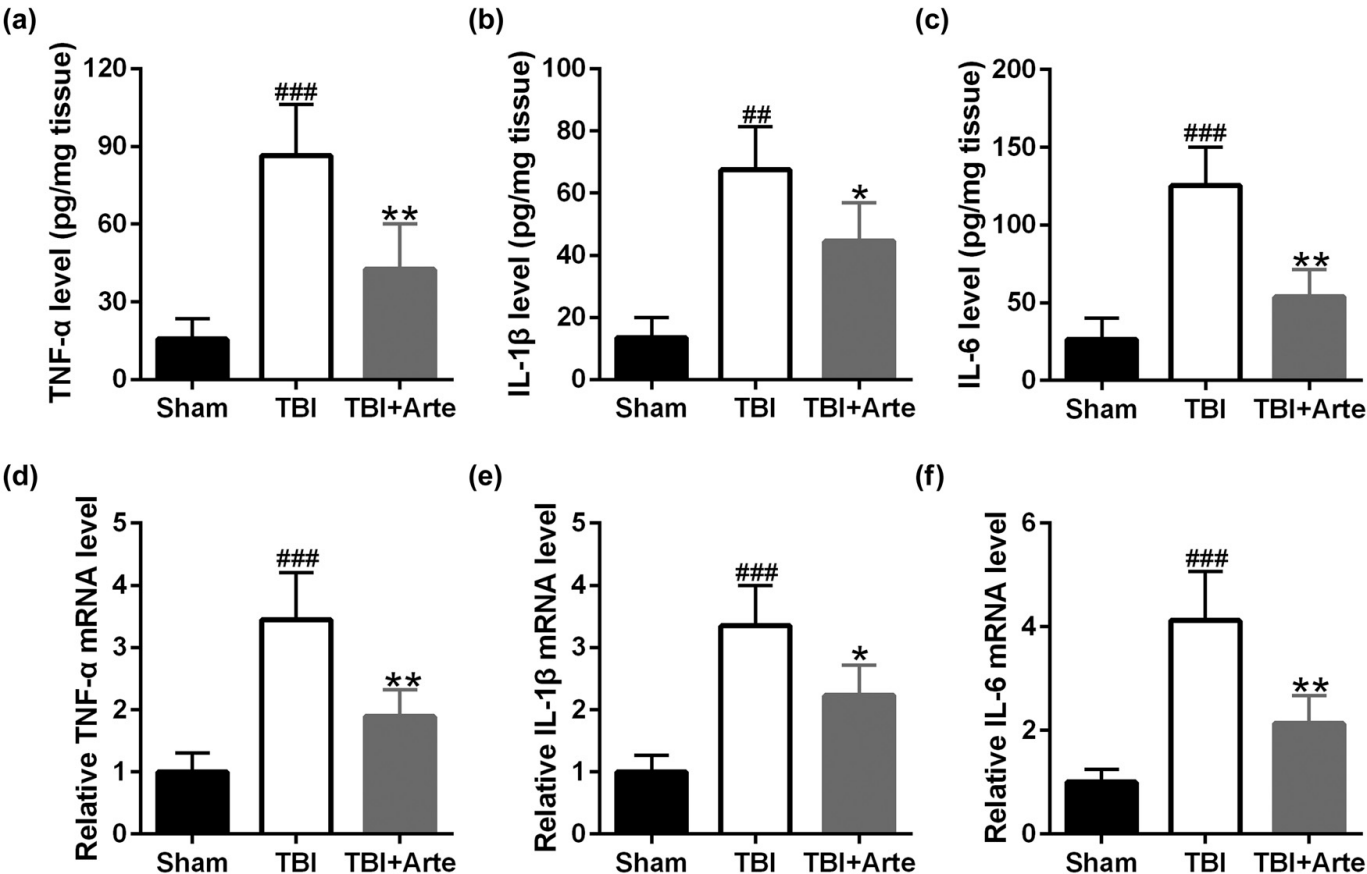
Artesunate inhibited traumatic brain injury-induced inflammatory response in the ipsilateral brain tissue of experimental rats 3 days post-injury. ELISA was used to analyze the levels of TNF-α (a), IL-1β (b) and IL-6 (c). qRT-PCR was used to analyze the mRNA levels of TNF-α (d), IL-1β (e) and IL-6 (f). Data are presented as mean ± SD. *n* = 8 for each group. ^##^ *p* < 0.01 and ^###^ *p* < 0.001 compared to sham group. *p < 0.05 and **p < 0.01 compared to the TBI group.

Increasing evidence has revealed that artesunate could inhibit the inflammation. To demonstrate that artesunate inhibited the neuroinflammatory response in TBI rats, ELISA was first performed. As shown in Figure 5a–c, the circulating TNF-α (A), IL-1β (B) and IL-6 (C) levels increased after the TBI and decreased dramatically in artesunate-treated rats. Similarly, the RNA levels of TNF-α (D), IL-1β (E) and IL-6 (F) were examined by qRT-PCRs, and artesunate treatment induced the downregulation of all these three chemokines in the brain tissue of TBI rats. These results suggested that artesunate inhibited neuroinflammation and thus improved the long-term neurological recovery of TBI rats.

#### 3.4.1 Artesunate regulated the phosphorylation levels of Akt and GSK-3β in TBI rats

**Figure 6:**
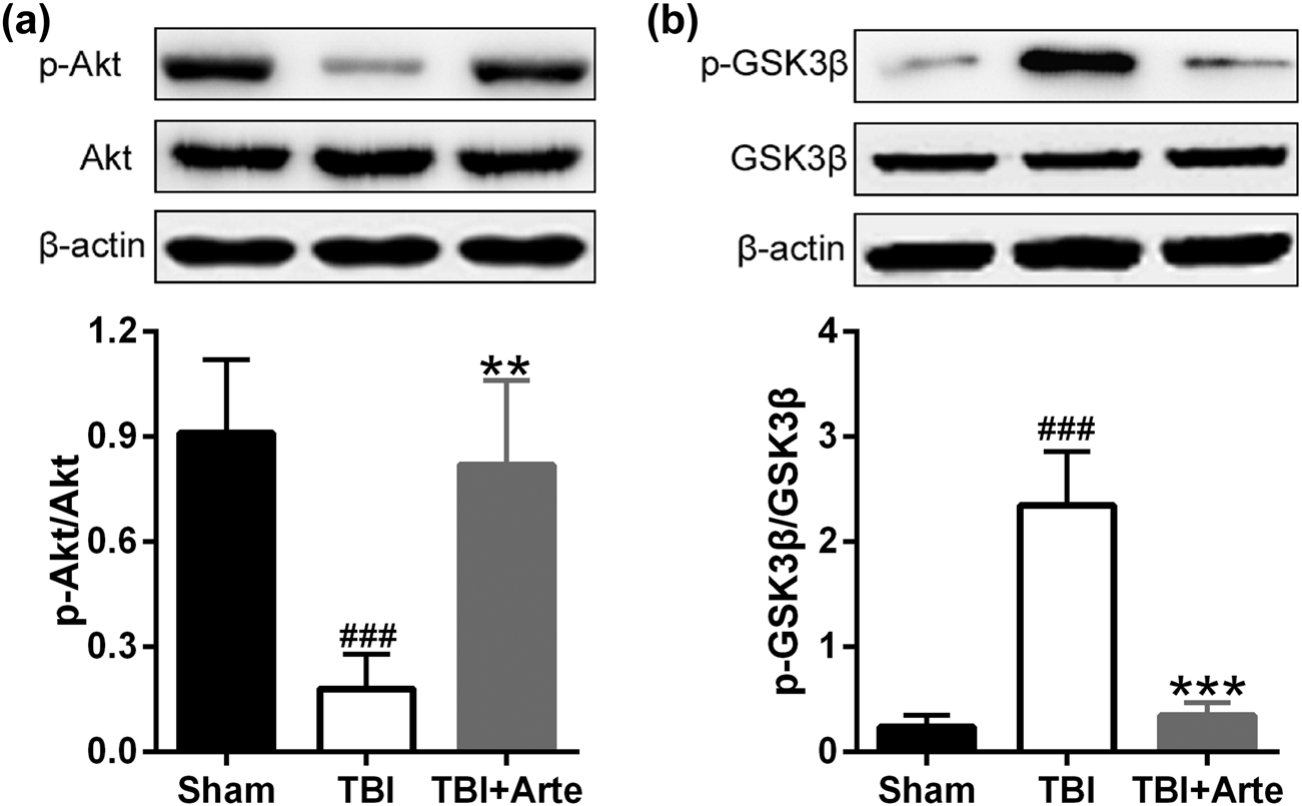
The expressions of Akt and GSK-3β in the ipsilateral brain tissue of experimental rats 3 days post-injury. Representative Western blot bands and quantitative analysis of the expressions of p-Akt/Akt (a) and p-GSK-3β/GSK-3β (b). Data are presented as mean ± SD. *n* = 8 for each group. ^###^ *p* < 0.001 compared to sham group. **p < 0.01 and ***p < 0.001 compared to TBI group.

To further identify the mechanism of artesunate to protect the brain tissue in TBI rats, the phosphorylation and expression levels of Akt and GSK-3β were examined. As shown in Figure 6a, the phosphorylation of Akt increased dramatically after the treatment of artesunate while there was no alternation in the total expression level of Akt. On the contrary, artesunate decreased the phosphorylation level of GSK-3β significantly after the TBI induction (Figure 6b). These two figures together demonstrate that artesunate attenuated brain damage in TBI, and this process was connected to the Akt and GSK-3β pathways.

## 4 Discussion

TBI remains a significant clinical challenge in spite of several breakthroughs of trauma management strategies in recent years [25]. The incidence of TBI is up to 2–3% in the industrialized countries in Europe, and it has been ranked as one of the leading causes of morbidity and mortality of adolescent and adults under 40 years of age all over the world [26]. The lasting and long-term syndrome composing of brain edema, cerebral infraction, intracranial hypertension, neural inflammation, necrosis, hydrocephalus, BBB dysfunction and so on makes TBI management more challenging and complex compared to the primary structure destruction caused by TBI itself [27]. However, the medical–surgical approaches combined with intensive care unit management for the treatment of TBI have altered very little over the past 30 years [28,29]. Given the high fatality rate and complex regulatory network of TBI, it is urgent for us to identify special targets or effective chemicals for the protective and restorative treatment. Here, we reported that artesunate protected the brain tissue from damage induced by TBI and improved the long-term neural recovery in TBI rat model. We demonstrated that treating the TBI rats with artesunate induced the accelerated recovery of neuron and motor functions and improved the recovery of memory and learning function loss in rats with brain trauma. In our study, we also demonstrated that artesunate treatment improved the integrity of BBB of TBI rats by upregulating the protein levels of occludin and Zo-1 and downregulating the expression levels of MMP-9. Besides, we identified artesunate treatment could inhibit the neural inflammatory response in the damaged brain tissue, which could be one of the most possible mechanisms of the attenuation induced by artesunate to TBI.

Artesunate is one of the most widely used antimalaria drugs and a semisynthetic derivative of artemisinin which was first distilled from a Chinese herb, Qinghao [30]. Although artesunate has been recommended by the WHO for the treatment of malaria, accumulating evidence has demonstrated the abundant potential applications of artesunate in the treatment of various diseases. For example, artesunate has been revealed to protect the normal tissues from hemorrhage and anoxia induced by organ trauma and dysfunction through phosphorylation (activation) of protein kinase B (Akt) and phosphorylation (inhibition) of glycogen synthase kinase-3β (GSK-3β) [30]. It has also been reported that artesunate could attenuate the fibrosis of hepatic cells by upregulating the microtubule-associated protein light chain 3 and downregulating ferritin heavy chain and nuclear receptor co-activator 4 [31]. Besides, drug screening assays have revealed that artesunate might function to treat cancer and research has demonstrated that artesunate could dramatically inhibit the proliferation of invasive tumor in mice model by upregulating the expression levels of DNA damage-inducible transcript 3 (DDIT3) and activating transcription factor 4 (ATF-4) factors which increased the endoplasmic reticulum stress and induced the apoptosis of malignant B cells [32,33].

Considering the multiple pharmacological functions of artesunate including anti-inflammation, anti-infection, antitumor and complex regulating mechanisms of artesunate to damaged tissues, research has realized that artesunate might be a potential candidate for the treatment of central nervous diseases since the symptoms and pathophysiologic mechanisms of them are numerous and complicated. For example, former studies have demonstrated that artesunate could regulate the expression of neurological factors and inhibit the proinflammatory reactions of TBI-induced cerebral lesions, thus promoting the recovery of neurons in the damaged areas and alleviating the symptoms caused by TBI [12,34]. Similarly, in our study, we also identified the cerebral neuron protective functions of artesunate to the dysfunctions induced by TBI. The MRI analysis identified that artesunate could alleviate cerebral lesions and reduce brain edema. The results of foot-fault assays and MWM tests both demonstrated that artesunate treatment could promote the recovery of cerebral neural dysfunction and accelerate the restoration of memory and motivation function in TBI rats.

BBB is composed of compact joint cerebrovascular endothelial cells, and the permeability of BBB plays a crucial role in maintaining the homeostasis of brain and systemic circulation. Brain edema, one of the most common secondary injuries induced by TBI, has been identified as the major cause of breakdown and dysfunction of BBB and thus induced unbalance in vascular and neural circulation [35]. Former research has revealed that artesunate could be used to treat brain edema. For instance, it has been reported that artesunate could alleviate brain edema and protect the integrity of BBB from SAH through S1P1 and PI3K pathways [17]. Occludin and Zo-1 played a significant role in the formation of tight junction of epithelium in BBB, and the expression levels of occludin and Zo-1 can be used to evaluate the integrity of BBB [23]. In our research, we identified that the treatment of artesunate to the TBI rats significantly upregulated the protein levels of occludin and Zo-1, thus demonstrating that artesunate could protect BBB from the dysfunction induced by TBI. Matrix metalloproteinase 9 (MMP-9) induced the degradation of BBB proteins, and its abnormal upregulation in the cerebral lesion regions contributes to the further destruction of BBB. In our study, we identified that artesunate treatment induced the downregulation of MMP-9 in the TBI areas, thus again indicating that artesunate could protect the integrity of BBB in TBI rat model.

GSK-3β and Akt are two crucial kinases and both participate in the regulation of numerous physiological activities [36]. The phosphorylation of Akt activated the kinase and inhibited the downstreamed inflammatory reactions, while the phosphorylation of GSK-3β inhibited its activity and raised the expression of claudin-3 and claudin-5, which are significant for maintaining the integrity and function of BBB [37]. Consistently, we identified that artesunate increased the phosphorylation level of Akt and reduced the phosphorylation level of GSK-3β in TBI rats, which further proved the protection of artesunate from the damaged BBB.

However, there are indeed some shortcomings in our research. First, due to resource and time constraints, the number of rats used in the study was limited. The conclusion could be more solid if more rats were studied. Second, when analyzing the integrity of the BBB, we mainly tested the expression levels of occludin and ZO-1. Some other indicators can also be introduced into our research to reflect more comprehensively the changes in BBB. We will continue to pay attention to these issues in our follow-up research.

In conclusion, we reported that artesunate treatment protected rats from TBI-induced impairments of BBB and improved longer-term neurological outcomes based on the existing evidence. Artesunate treatment attenuated the impact caused by TBI to rat brain and improved the long-term neurological recovery. Artesunate treatment protected the integrity of BBB and inhibited neuroinflammation. Artesunate treatment promoted the phosphorylation of Akt and inhibited the phosphorylation of GSK-3β in TBI rat model. We hope our research could provide a new strategy for the treatment of TBI in clinic and further revealed the complicated pharmacological mechanisms of artesunate.

## Abbreviations

BBB: blood–brain barrier
TBIs: traumatic brain injuries
CCI: cortical impact injury
